# Changes in body mass index and hemoglobin concentration in breastfeeding women living with HIV with a CD4 count over 350: Results from 4 African countries (The ANRS 12174 trial)

**DOI:** 10.1371/journal.pone.0177259

**Published:** 2017-05-09

**Authors:** Eric Nagaonlé Somé, Ingunn M. S. Engebretsen, Nicolas Nagot, Nicolas Y. Meda, Roselyne Vallo, Chipepo Kankasa, James K. Tumwine, Mandisa Singata, Justus G. Hofmeyr, Philippe Van de Perre, Thorkild Tylleskär, for the ANRs 12174 Trial Group

**Affiliations:** 1 Centre for International Health, University of Bergen, Bergen, Norway; 2 National Health Research Institute, Centre National pour la Recherche Scientifique et Technologique, Ouagadougou, Burkina Faso; 3 INSERM UMR 1058, Pathogenesis and control of chronic infections, Montpellier, France; 4 Université de Montpellier, Montpellier, France; 5 Centre Hospitalier Universitaire, Montpellier, France; 6 University of Ouagadougou, Faculty of Health Sciences, Centre de Recherche International en Santé (CRIS) Ouagadougou, Burkina Faso; 7 University of Zambia, School of Medicine, Department of Paediatrics and Child Health, University Teaching Hospital, Lusaka, Zambia; 8 Makerere University, Department of Paediatrics and Child Health, School of Medicine, College of Health Sciences, Kampala, Uganda; 9 University of Fort Hare, Effective Care Research Unit, Eastern Cape, South Africa; University of California, San Francisco, UNITED STATES

## Abstract

**Introduction:**

Breastfeeding is recommended for infants born to HIV-infected women in low-income settings. Both breastfeeding and HIV-infection are energy demanding. Our objective was to explore how exclusive and predominant breastfeeding changes body mass index (BMI) among breastfeeding HIV1-positive women participating in the ANRS12174 trial (clinical trial no NCT0064026).

**Methods:**

HIV-positive women (n = 1 267) with CD4 count >350, intending to breastfeed HIV-negative infants were enrolled from Burkina Faso, South Africa, Uganda and Zambia and counselled on breastfeeding. N = 1 216 were included in the analysis. The trial compared Lamivudine and Lopinavir/Ritonavir as a peri-exposure prophylaxis. We ran a linear mixed-effect model with BMI as the dependent variable and exclusive or predominant breastfeeding duration as the key explanatory variable.

**Results:**

Any breastfeeding or exclusive/predominant) breastfeeding was initiated by 99.6% and 98.6% of the mothers respectively in the first week after birth. The median (interquartile range: IQR) duration of the group that did any breastfeeding or the group that did exclusive /predominant breastfeeding were 9.5 (7.5; 10.6) and 5.8 (5.6; 5.9)) months, respectively. The median (IQR) age, BMI, CD4 count, and HIV viral load at baseline (day 7) were 27 (23.3; 31) years, 23.7 (21.3; 27.0) kg/m^2^, 530 (432.5; 668.5) cells/μl and 0.1 (0.8; 13.7)1000 copies/mL, respectively. No major change in mean BMI was seen in this cohort over a 50-week period during lactation. The mean change between 26 and 50 weeks after birth was 0.7 kg/m^2^. Baseline mean BMI (measured on day 7 postpartum) and CD4 count were positively associated with maternal BMI change, with a mean increase of 1.0 kg/m^2^ (0.9; 1.0) per each additional baseline-BMI kilogram and 0.3 kg/m^2^ (0.2; 0.5) for each additional CD4 cell/μl, respectively.

**Conclusion:**

Breastfeeding was not negatively correlated with the BMI of HIV-1 infected Sub-Saharan African mothers. However, a higher baseline BMI and a CD4 count >500 cells/μl were associated with maternal BMI during the exclusive/ predominant breastfeeding period. Considering the benefits of breast milk for the infants and the recurrent results from different studies that breastfeeding is not harmful to the HIV-1-infected mothers, this study also supports the WHO 2016 guidelines on infant feeding that mothers living with HIV should breastfeed where formula is not safe for at least 12 months and up to 24 months, given that the right treatment or prophylaxis for the infection is administered. These findings and conclusions cannot be extrapolated to women who are immune-compromised or have AIDS.

## Introduction

HIV infection is among the leading causes of mortality among women of childbearing age [1]. It will be associated with an increased risk of infections from non-obstetric and direct obstetric causes, making HIV-infected mothers more vulnerable than uninfected mothers [2,3]. HIV in pregnancy contributed to high maternal mortality rates with a 9% contribution in Sub-Saharan Africa (SSA) between 1990 and 2008. It is the leading cause of death during pregnancy and the postpartum period in countries with a high prevalence of HIV [4,5]. A recent meta-analysis pointed out clearly the significance of HIV infection in global maternal mortality rates, with an estimated 5% pregnancy-related deaths worldwide and 25% in SSA [6]. The reasons of this high mortality in HIV-infected women are unclear. The risk of obstetric complications may be increased in HIV-infected women or pregnancy might accelerate HIV progression [6–8].

Weight loss and low body mass index (BMI) can serve as markers of HIV disease progression. Weight has been used to diagnose clinical AIDS disease; a 10% weight loss in the absence of any other evident cause was one of the early WHO clinical criteria in areas without laboratories [9–13]. Individuals with asymptomatic HIV need an extra 10% energy intake to maintain body weight, which increases to 20–30% among those who have symptomatic HIV [14,15]. Moreover during pregnancy and lactation, the woman’s body in a normal physiological state undergoes massive energy trade-off seen through weight changes, typically an increase during pregnancy and a loss to pre-pregnancy weight during lactation [16,17].

There has been a dilemma during the last 20 years in choosing between exclusive breastfeeding (EBF) and replacement feeding [18], which was only considered on the basis of the best nutritional and survival outcome for the infant. Specifically, in the pre-ART period the focus was on ensuring the HIV-free survival of infants exposed to HIV.However, in socio-economically deprived settings, breastfeeding has proven to be a key survival strategy for infants born to women living with HIV because increased morbidity and mortality have been associated with replacement feeding [19]. After 2010, availability of, and accessibility to, antiretroviral drugs during pregnancy and lactation have increased. The programs of prevention of mother-to-child transmission (PMTCT) of HIV yielded 2 antiretroviral (ARV) prophylaxis options, including regimen A (prophylactic ARV drugs are given to the mothers and children during the risk period); and regimen B (antiretroviral therapy is given to the mothers during the risk period), or regimen B+ (extending regimen B to lifelong treatment) [20]. In the WHO’s 2010 recommendations, women living with HIV who opted to breastfeed were recommended to practice exclusive breastfeeding (EBF) until the infant was 6 months old, introduce appropriate complementary foods thereafter, and continue breastfeeding for the first 12 months of the infant’s life. The 2016 recommendations recommend mothers living with HIV to prolong the breastfeeding period to up to 24 months, as advised for the general population (i.e. HIV-uninfected women). The importance of having a nutritionally adequate and safe diet for the child, a lifelong ARV therapy, with adherence counselling and support for breastfeeding for the mothers, is stressed as a prerequisite before breastfeeding can be gradually stopped. Thus, option B+ has made it easier for countries to opt for exclusive breastfeeding for 6 months as the preferred early child feeding option [21–23].

Regarding the mother’s health, studies have reached inconsistent conclusions as to the effect of breastfeeding on the weight of those infected with HIV [14,24,25]. The South African Vertical Transmission Study that compared women practicing EBF compared to those opting for replacement feeding or mixed feeding concluded that a) HIV-infected and uninfected mothers experienced similar weight loss over 24 months; and b) postpartum weight change was not associated with feeding practices for the first 5 months of the baby’s life. [14]. In this study, the mothers received a single-dose of nevirapine as PMTCT treatment. The Kesho Bora study, a combination of a randomized clinical trial and a prospective cohort study, comprised 3 parts: part IA included women with CD4 count <200 cells/mm^3^, receiving Zidovudine (AZT), lamivudine and nevirapine (NVP) twice daily; part IB included women with CD4 count >500 cells/mm^3^, receiving AZT 300 mg taken by the mother twice daily starting from 34 to 36 weeks of pregnancy until the onset of labor, plus one 600 mg dose of AZT and one 200 mg dose of NVP at the onset of labor; and part II or the RCT part, included women with a CD4 count between 200 and 500 cells/mm^3^, randomized to receive the same prophylaxis as part IB or triple-ARV prophylaxis (AZT [300 mg], 3TC [150 mg] and lopinavir/ritonavir [LPV/r, 400 mg/100 mg]) twice daily from 34 to 36 weeks of pregnancy, through delivery and during breastfeeding to a maximum of 6 months postpartum [26]. It showed that 6 months of breastfeeding was not detrimental to the weight of well-nourished HIV-infected mothers [24]. A study in Zambia randomized women into a short-duration (4 months EBF and abrupt weaning) and a long-duration (mean duration of 16 months including 6 months EBF) breastfeeding period; it showed a net weight gain in HIV-infected women breastfeeding from 4 up to 24 months postpartum [27]. All mothers in the Zambian study were categorized into low (≤350 cells/μl) and high (>350 cells/ μl) CD4 count groups. The subjects had received a single-dose nevirapine as MTCT prophylaxis. When antiretroviral treatment became available in May 2004, 26 women were started on the first-line regimen. CD4 count was associated with lower weight. Effects of lactation in women with low CD4 counts were similar to the effects in women with higher CD4 counts. In contrast, two Kenyan studies [28,29] found weight loss among HIV-infected breastfeeding mothers compared to mothers using formula feeding. Though the design of these studies made a comparison difficult with the precedent studies. they were worse in showing adverse outcomes due to breastfeeding, which included more maternal deaths. These studies in Nairobi, representing two different analyses of the same data, compared mothers who breastfed from delivery until 2 years of age to formula-feeding in a cohort study where all the pregnant women received a short course of zidovudine prophylaxis. When antiretroviral treatment became available, women were referred to highly active antiretroviral treatment programs.

HIV infection also seems to be detrimental to maternal hemogloblin concentration [30,31], which could be worsened by the use of some antiretroviral substances [32–34]. Our aim was therefore to explore within the ANRS12174 trial population how breastfeeding might change BMI and hemoglobin concentration. We also assessed other factors that might have influenced weight changes of HIV-infected mothers during lactation.

## Methods

### Study design

The ANRS 12174 clinical trial in Ouagadougou (Burkina Faso), East London (South Africa), Mbale (Uganda) and Lusaka (Zambia) was conducted from 2009 to 2013, the protocol and the primary analysis having been published [35,36]. Briefly, pregnant women who tested positive for HIV-1 infection in the context of routine antenatal clinic service making them ineligible for highly active antiretroviral therapy because their CD4+ count was >350 cells/ml, who had to be at least 18 years old and who were planning to breastfeed, were identified at antenatal clinics between 28 and 40 weeks of pregnancy. They received a pre-test counselling session before testing for HIV infection. As part of the post-test session, they were informed of the different feeding options for their babies. Only women intending to breastfeed were referred to the research clinic for further assessment of the inclusion criteria during the antenatal period, and again with their child within 6 days after birth, for enrolment and randomisation at day 7 postpartum. From 28 weeks of pregnancy to day 7 after birth, programmatic mother-to-child transmission prophylaxis was followed with antepartum zidovudine, intrapartum single dose nevirapine, and zidovudine-lamivudine for mothers and nevirapine for infants for 7 days postpartum. The intervention implemented during the trial was infant prophylaxis in the breastfeeding period starting from 7 days to 50 weeks of age with either lopinavir/ritonavir or lamivudine. Twins and triplets, infants with a positive HIV-1 DNA PCR test result at day 7 (+/- 2) postpartum, and low birth-weight or ill babies (ranked grade II or above of the ANRS classification for adverse events) on the day of enrolment were excluded [37].

Lamivudine, generally well tolerated and accepted, has been widely used in research and clinical trials. The lopinavir/ritonavir paediatric formulation has been a very promising prophylactic combination with low risks for resistance, high antiviral potency and a good safety profile [35]. However, it is known to be distasteful (https://www.medicines.org.uk/emc/medicine/4602; https://aidsinfo.nih.gov/contentfiles/lvguidelines/adultandadolescentgl.pdf), which was thought to matter less when introduced very early.

### Data management and analysis

Data were collected on a paper case-report form or directly entered online using the Electronic Data capture system OpenClinica™ (www.openclinica.com). Twenty-four hours and one week breastfeeding recalls were collected during the enrolment visit on day 7 (±2) after birth and during the 13 monthly-scheduled follow-up visits that started at week 2. During these visits, mothers were particularly asked if they gave their infants other foods/liquids in addition to breastmilk. Pre-lacteal feeding data which was defined as any food item except mothers’ milk given to infants before initial breastfeeding) were also collected at the enrolment visit. The data were collected by trained physicians, pharmacists, biologists and counsellors. Seca-brand scales and stadiometers were used to measure the mother’s height and weight. Weights were rounded to the nearest 10 grams and the height at the nearest millimetre. Weight and height were measured twice based on the WHO guidelines (http://www.who.int/childgrowth/training/en/).

We categorized mothers at each visit into the following groups: 1) exclusive breastfeeding, EBF (only breastmilk being given to the infant without any other kind of food or liquid, except medically prescribed drugs or vitamins); 2) predominant breastfeeding, PBF (breastmilk with some liquid-based food, such as juice, tea, sugar-water and salt-water including glucose without any kind of formula or animal milk); and 3) mixed feeding, MF (breastmilk with other solid or liquid-based food, including other kinds of milk). We thereafter combined EBF and PBF into one group called “exclusive and predominant breastfeeding” (EPBF) because the number of women who practised PBF was too small and the practice was occasional. The entire cohort was in this latter group at the beginning of the study, which was followed up to detect any change in EPBF status, i.e. change to mixed feeding by week 26 post-partum, which is the time when exclusive breastfeeding is supposed to be changed to complementary feeding. Data on maternal dietary intake were not collected

In a bivariate analysis, we compared the mean BMI at weeks 14, 26, 38 and 50 with the mean BMI at day 7 (baseline) post-delivery using Bonferroni-corrected paired t-tests for multiple comparisons. Data were presented for all 4 sites, by country and baseline BMI groups. Group 1 included women with BMI<18.5 kg/m^2^ (underweight), Group 2 included those with a BMI between 18.5 and 24.9 kg/m^2^ (normal range), and Group 3 were those with a BMI ≥25 kg/m^2^ (overweight) according to the WHO classification system (http://apps.who.int/bmi/index.jsp?introPage=intro_3.html) [38]. We also compared the mean hemoglobin concentration at week 38 with week 14 (because this data was available only for these visits) in the same way. To take into account the inter-country and inter-subject variability, we ran a further linear mixed-effect model with BMI as the dependent variable and EPBF duration as the key explanatory variable adjusted for baseline BMI and other covariates, including the mother’s viral load, CD4 count, hemoglobin concentration, HIV stage, mode of delivery (vaginal versus C-section), parity, age, education level, marital status and occupation. The child’s characteristics included its gender, birth weight, treatment group and the breastfeeding initiation time. We checked that the total variance due to country effect was always >10% by running a variance component analysis and a likelihood ratio test to confirm that the differences related to countries were significant. Variables with p≤0.20 in the bivariate analysis were considered for the multivariate analysis in the pooled-data analysis for all the countries. We also stratified by country and ran the same multivariate analysis, introducing a country-specific socio-economic status (SES) index.

The principal component methods was used to construct the SES index, [39]. Sixteen asset variables were variously included in the principal component analysis, considering country specificities. The first components (explaining 33, 39, 29 and 34% of the variation for Burkina Faso, South Africa, Uganda and Zambia, respectively) were retained to weigh the variables and calculate the index at the household level. This was the sum of the different variables’ weight/score per subject, which was divided into tertiles.

For continuous variables, the mean values with 95% confidence interval (CI) were estimated, and for categorical variables, percentages were used. Medians (IQR) were also reported. Associations between variables were tested using the Chi-square test for categorical variables. STATA/SE 13.1 (4905 Lakeway Drive College Station, Texas 77845 USA) was the statistical software used.

### Ethics

Prior to enrolment, the mothers signed written informed consent and assent forms for themselves and their children, respectively. The trial was conducted according to the sponsor (ANRS) ethic charter, Good Clinical Practices and the principles of the Helsinki declaration. The protocol had obtained approval from the relevant ethical committees, including the Ethical Committee for Health Research in Burkina Faso, the Biomedical Research Ethics Committee in Zambia, the Uganda National Council for Science and Technology, the Stellenbosch University ethical committees, the Medicines Control Council in South Africa and the Regional Committee for Medical Research Ethics of Norway.

## Results

In the ANRS 12174 trial, 1,273 mother-infant pairs were randomized and 6 were excluded due to protocol violations. Of the remaining 1,267 participants, 204 were from Ouagadougou, 222 from East London, 278 from Mbale and 563 from Lusaka. In all, 42 were excluded from analysis due to lack of breastfeeding data after inclusion, 7 due to inaccurate feeding duration data and 2 women had no data on weights. Thus 1,216 subjects were included in the analysis.

At baseline (Table 1), South Africa had the largest mean BMI, and the highest frequency of single women and C-section delivery. Burkina Faso participants had the lowest HIV viral load, the lowest hemoglobin concentration, and the lowest literacy and formal occupation frequency. Breastfeeding was initiated later in Burkina Faso where EPBF frequency was also the lowest the first week.

**Table 1:** Baseline characteristics.

**A**	**Burkina Faso**	**South Africa**	**Uganda**	**Zambia**	**All sites**
	**N = 203**	**N = 212**	**N = 272**	**N = 529**	**N = 1216**
	**% (95% CI**^**a**^**)**	**% (95% CI**^**a**^**)**	**% (95% CI**^**a**^**)**	**% (95% CI**^**a**^**)**	**% (95% CI**^**a**^**)**
CD4>500	57.1 (50.2; 63.8)	51.4 (44.7; 58.1)	57.3 (51.4; 63.1)	59.9 (55.7; 64.0)	57.4 (54.6; 60.2)
Education level					
Incomplete primary	68.5 (61.7; 74.5)	8.5 (5.4; 13.1)	48.5 (42.6; 54.5)	28.2 (24.5; 32.2)	36.0 (33.4; 38.8)
Completed primary	7.4 (4.5; 11.9)	0.5 (0.1; 3.3)	15.8 (11.9; 20.6)	18.5 (15.4; 22.1)	12.9 (11.1; 14.9)
Secondary and more	24.1 (18.7; 30.5)	91.0 (86.4; 94.2)	35.7 (30.2; 41.5)	53.3 (49.0; 57.5)	51.1 (48.2; 53.9)
Occupation (employed)	8.9 (5.6; 13.6)	41.5 (35.0; 48.3)	35.3 (29.8; 41.2)	17.0 (14.0; 20.5)	24.0 (21.7; 26.5)
Married/co-habiting	90.6 (85.8; 93.0)	39.1 (32.8; 45.9)	82.0 (76.9; 86.1)	88.7 (85.7; 91.1)	78.9 (76.5; 81.1)
Mode of delivery (vaginal)	93.6 (89.3; 96.2)	65.1 (58.4; 71.2)	93.4 (89.7; 95.8)	96.2 (94.2; 97.5)	89.7 (87.9; 91.3)
Parity (primiparous)	21.7 (16.5; 27.9)	33.5 (27.4; 40.1)	18.0 (13.9; 23.0)	20.6 (17.4; 24.3)	22.4 (20.2; 24.9)
Trial arm (lamivudine)	49.7 (42.9; 56.6)	51.9 (45.1; 58.6)	49.6 (43.7; 55.6)	50.3 (46.0; 54.5)	50.3 (47.5; 53.1)
HIV stage 1	93.1 (88.7; 95.9)	98.6 (95.7; 99.5)	92.3 (88.4; 94.9)	99.8 (98.7; 100.0)	96.8 (95.6; 97.6)
Child sex (male)	41.9 (35.2; 48.8)	49.1 (42.4; 55.8)	52.9 (47.0; 58.8)	48.4 (44.1; 52.7)	48.4 (45.6; 51.2)
ART prophylaxis postpartum	100.0	7.1 (4.3; 11.4)	71.0 (65.3; 76.1)	100.0	77.3 (74.9; 79.6)
Breastfeeding initiation time					
Within 1^st^ hour	6.9 (4.1; 11.3)	51.4 (44.7; 58.1)	55.9 (49.9; 61.7)	80.7 (77.1; 83.9)	57.7 (54.9; 60.5)
After 1^st^ hour and within 1^st^ day	64.0 (57.2; 70.4)	45.7 (39.1; 52.5)	41.2 (35.5; 47.1)	18.9 (15.8; 22.5)	36.1 (33.4; 38.8)
After 1^st^ day	29.1 (23.2; 35.7)	2.8 (1.3; 6.2)	2.9 (1.5; 5.8)	0.4 (0.1; 1.5)	6.2 (4.9; 7.7)
EPBF^b^ 1^st^ 3 days	93.1 (88.7; 95.9)	94.3 (90.3; 96.8)	97.8 (95.2; 99.0)	99.0 (97.7; 99.6)	97.0 (95.8; 97.8)
EPBF^b^ last 4 days	94.1 (89.8; 96.6)	95.7 (92.1; 97.8)	97.4 (94.7; 98.8)	99.6 (98.5; 99.9)	97.5 (96.5; 98.3)
Any BF 1^st^ week	100.0	97.6 (94.4; 99.0)	100.0	100.0	99.6 (99.0; 99.8)
EPBF^b^ 1^st^ week	96.5 (92.9; 98.3)	97.2 (93.8; 98.7)	98.9 (96.6; 99.6)	99.8 (98.7;100.0)	98.6 (97.8; 99.1)
SES^c^ tertiles					
Highest tertile	31.5 (30.3 32.8)	19.8 (18.8 20.9)	38.2 (37.1 39.3)	42.3 (41.5 43.1)	
Middle tertile	39.4 (38.1 40.7)	73.6 (72.4 74.7)	27.9 (26.9 29.0)	17.4 (16.8 18.0)	
Lowest tertile	29.1 (27.9 30.3)	6.6 (6.0 7.3)	33.8 (32.7 34.9)	40.3 (39.5 41.1)	
**B**					
	**N = 203**	**N = 212**	**N = 272**	**N = 529**	**N = 1216**
	**Median (IQR**^**d**^**)**	**Median (IQR**^**d**^**)**	**Median (IQR**^**d**^**)**	**Median (IQR**^**d**^**)**	**Median (IQR**^**d**^**)**
Maternal age (years)	28.0 (24.3; 32.0)	27.3 (23.5; 32.8)	26.9 (23.0; 29.9)	26.8 (23.3; 31.0)	27.0 (23.3; 31.0)
BMI (kg/m^2^)	23.0 (21.2; 25.4)	27.8 (24.2; 31.8)	22.8 (20.8; 24.6)	23.2 (21.2; 26.5)	23.7 (21.3; 27.0)
CD4+ cells/μL	526 (428; 653)	505 (426; 648.5)	524.5 (429.5; 624)	548 (445; 705)	530 (432.5; 668.5)
Gestational age (weeks)	39.0 (37.0; 40.0)	38.0 (38.0; 38.0)	40.0 (39.0; 40.0)	38.0 (37.0; 40.0)	38.0 (38.0; 40.0)
HIV viral load^e^ (x1000 /mL)	1.8 (0.7; 7.2)	2.1 (0.8; 7.9)	4.1 (0.6; 17.5)	3.8 (1.2; 20.6)	3.0 (0.8; 13.7)
Haemoglobin (g/dL)	11.2 (10.5; 12.1)	12.0 (11.3; 12.9)	12.4(11.7; 13.2)	12.5 (11.6; 13.3)	12.2 (11.3; 13.1)
Birth weight (kg)	2.9 (2.7; 3.2)	3.2 (2.8; 3.5)	3.0 (2.8; 3.3)	3.0 (2.8; 3.3)	3.0 (2.8; 3.3)

^**a**^ Confidence Interval

^b^ Exclusive and predominant breastfeeding

^c^ Socio-economic status

^**d**^ Interquartile range

^e^ N = 173, 167, 180, 477 and 997 for Burkina Faso, South Africa, Uganda, Zambia and overall, respectively

^f^ N = 175, 161, 218, 454 and 1008 for Burkina Faso, South Africa, Uganda, Zambia and overall, respectively

During pregnancy antiretroviral prophylaxis was given 100% of women in all 4 countries

### Breastfeeding duration and BMI changes

The median (Interquartile Range (IQR)) durations of EPBF and any breastfeeding were 5.8 (5.6; 5.9) and 9.5 (7.5; 10.6) months, respectively. The median (IQR) durations of EPBF were 20.9 (20.0; 21.5), 19.8 (12.9; 21.0), 20.9 (19.9; 21.0), 21.0 (20.6; 21.1) for Burkina Faso, South Africa, Uganda and Zambia, respectively

The BMI of breastfeeding mothers decreased from baseline to week 26 before plateauing until week 50 (Table 2). The same linear trend in the association between breastfeeding duration and BMI decrease was found in the country-specific analysis except in Uganda where we did not find any significant decrease. The maximum weight loss was at week 26 (p<0.001) in Burkina Faso and Zambia, whereas it was at week 38 for South Africa and Uganda.

**Table 2:** Country-stratified paired t-test comparing mean BMI at different endpoints with baseline mean BMI.

	Burkina Faso	South Africa	Uganda*	Zambia	All sites
	Mean difference (kg/m^2^) (95% CI^a^)	Mean difference (kg/m^2^)(95% CI^a^)	Mean difference (kg/m^2^) (95% CI^a^)	Mean difference (kg/m^2^)(95% CI^a^)	Mean difference (kg/m^2^)(95% CI^a^)
BMI at week 14 minus BMI at baseline (D7)	-0.8 (-1.1; -0.4) §	-0.8 (-1.4; -0.3) §	-0.2 (-0.5; 0.1)	-0.5(-0.8; -0.2) §	-0.5(-0.7; -0.4)§
BMI at week 26 minus BMI at baseline (D7)	-1.1 (-2.4; 0.2)	-0.8 (-1.5; -0.1) §		-0.6(-0.9; -0.2) §	-0.7 (-1.0; -0.3)§
BMI at week 38 minus BMI at baseline (D7)	-0.9 (-1.5; -0.4) §	-0.9 (-1.8; -0.0) §	-0.4 (-0.9; 0.1)	-0.6(-1.0; -0.2) §	-0.7 (-0.9; -0.4)§
BMI at week 50 minus BMI at baseline (D7)	-1.0 (-1.7; -0.2) §	-0.8 (-1.7; 0.1)		-0.6(-1.0; -0.2) §	-0.7 (-1.0; -0.3)§

* data on mothers’ weight were missing on weeks 26 and 50 in Uganda

§ Significant results

Comparing the hemoglobin concentration at weeks 14 and 38, there was no overall change, as also in the country-stratified analysis (p>0.05; see Table 3).

**Table 3:** Country-stratified paired t-test comparing mean hemoglobin at week 14 and week 38 (hemoglobin at week 14 > hemoglobin at week 38).

	Burkina Faso	South Africa	Uganda	Zambia	All sites
Mean hemoglobin (g/dl) at W14 (95% CI)	11.2 (10.8; 11.7)	12.0 (11.5; 12.5)	12.4 (12.1; 12.8)	12.4 (12.1; 12.7)	12.1 (11.9; 12.3)
Mean hemoglobin (g/dl) at W38 (95% CI)	11.2 (10.7; 11.6)	12.1 (11.6; 12.6)	12.5 (12.2; 12.9)	12.6 (12.3; 12.8)	12.3 (12.1; 12.5)
Mean difference (g/dl) (95% CI)	-0.1 (-0.5; 0.3)	0.1 (-0.3; 0.5)	0.1 (-0.2; 0.4)	0.2 (-0.1; 0.5)	0.1 (-0.1; 0.3)

Categorizing women into 3 BMI groups, we found that slimmer women (BMI <18.5 kg/m2) had no statistically significant change in their BMI postpartum (p>0.05), while the others had significant decrease over the breastfeeding period (Table 4). The thinnest group of women (BMI <18.5) had the steepest drop in hemoglobin at week 38; however, the difference was not significant (-0.3 (-0.8; 0–3); see Table 5).

**Table 4:** Women stratified in 3 BMI-categories: Paired t-test comparing mean BMI at different endpoints with baseline mean BMI.

	BMI<18.5 N = 50	BMI between 18.5 and 24.9 N = 715	BMI≥25 kg N = 451	All categories N = 1,216
	Mean difference (kg/m^2^) (95% CI^a^)	Mean difference (kg/m^2^) (95% CI^a^)	Mean difference (kg/m^2^) (95% CI^a^)	Mean difference (kg/m^2^) (95% CI^a^)
BMI at week 14<BMI at baseline (D7)	-0.0 (-1.1; 1.0)	-0.4 (-0.6; -0.2)§	-0.8 (-1.1; -0.4)§	-0.5 (-0.7; -0.4)§
BMI at week 26<BMI at baseline (D7)	-0.2 (-1.6; 1.2)	-0.6 (-1.0; -0.2)§	-0.8 (-1.3, -0.2)§	-0.7 (-1.0; -0.3)§
BMI at week 38<BMI at baseline (D7)	-0.1 (-1.2; 0.9)	-0.7 (-0.9; -0.4)§	-0.8 (-1.3; -0.2)§	-0.7 (-0.9; -0.4)§
BMI at week 50<BMI at baseline (D7)	-0.5 (-2.1; 1.1)	-0.7 (-1.2; -0.3)§	-0.6 (-1.2; -0.0)§	-0.7 (-1.0; -0.3)§

§ Significant results

**Table 5:** BMI-categories-stratified paired t-test comparing mean hemoglobin at weeks 14 and 38 (hemoglobin at week 14> hemoglobin at week 38).

	Number of observations	Mean hemoglobin (g/dl) at W14 (95% CI)	Mean hemoglobin (g/dl) at W38 (95% CI)	Mean difference (g/dl) (95% CI)
**BMI<18.5**	36	12.1 (10.9; 13.3)	11.8 (10.7; 13.0)	-0.3(-1.5; 0.9)
**BMI between 18.5 and 24.9**	502	12.2 (11.9; 12.5)	12.3 (12.0; 12.6)	0.1 (-0.2; 0.3)
**BMI≥25 kg**	295	12.1 (11.8; 12.42)	12.3 (12.0; 12.6)	0.2 (-0.1; 0.5)

§ Significant results

### Factors associated with BMI changes in mothers

Overall, the univariate analysis showed that EPBF duration and all other controlled variables were significantly associated with the BMI change, but not with the CD4 count, possibly because the most vulnerable group, i.e. those with CD4 counts <350, were excluded from the analysis. However, this variable was kept in the final model because of the known association between CD4 count and HIV disease progression, usually leading to weight loss (Table 6). In the multivariate analysis shown in Table 6, no association between EPBF duration and mothers’ BMI was found. Only baseline BMI, CD4 counts, education level, marital status and child birth weight remained significant risk factors for BMI changes. Each additional kilogram of mother’s baseline weight increased BMI by 1.0 kg/m^2^ during the lactation period.

**Table 6:** Unadjusted and adjusted mixed-effect model analysis testing BMI change over duration of extended EBF.

	Unadjusted Coefficient (95% CI)	Adjusted Coefficient (95% CI)
**EPBF duration**	-0.1 (-0.1; -0.0)	-0 (0; 0)
**Baseline BMI**	1.0 (0.9; 1.0) §	1.0 (0.9; 1.0) §
**Mother’s age**	0.2 (0.1; 0.2) §	0 (0; 0)
**CD4 count**		
<500	1	1
> = 500	0 (0; 0)	0.3 (0.2; 0.5) §
**Number of children**	0.4 (0.3; 0.5) §	-
**Number of child death**	-1.0 (-1.5; -0.5) §	-
**BF initiation time**	0 (0; 0)	
Within 1 hour	1	-
After 1 hour and within 1^st^ day	-0.1 (-0.4; 0.1)	-
After 1^st^ day	0.9 (0.3; 1.5) §	-
**Child gender**		
Male	1	-
Female	0.2 (0.0; 0.5)	-
**HIV stage**		
1	1	1
>1	-1.1 (-1.8; -0.3) §	-0.1 (-0.4; 0.2)
**Education level**	0.3 (0.1; 0.5) §	
No education or some primary	1	1
Complete primary	1.0 (0.6; 1.5) §	-0.3 (-0.4; -0.1) §
Secondary and more	0.6 (0.3; 1.0) §	-0.1 (-0.3; -0.0)
**Marital status**		
Single	1	1
Married/cohabiting	-0.9 (-1.2; -0.6) §	-0.3 (-0.4; -0.2) §
**Mode of delivery**		
Vaginal	1	-
C-section	1.8 (1.4; 2.1) §	-
**Parity**		
Primiparous	1	-
Multiparous	1.8 (1.5; 2.0) §	-
**Trial arm**		
lamivudine	1	1
lopinavir/ritonavir	0.2 (0.0; 0.4)	-0.0 (-0.1; 0.0)
**Child birth weight**	0 (0; 0)	
<2.5 kg	1	1
2.5 to 3.4 kg	1.0 (0.5; 1.4) §	-0.0 (-0.2; 0.2)
> = 3.5 kg	3.2 (2.8; 3.7) §	0.2 (0.0; 0.4)
**Gestational age**	0.5 (0.4; 0.6) §	-

§ Significant results

Compared to mothers with a CD4 count of 350–500 cells/μl, the group with the higher count had an increase in their BMI of 0.3 kg/m^2^. Regarding education level, the group that completed primary and secondary or further education had a reduced BMI of 0.3 and 0.1 kg/m^2^, respectively, over the lactation period. Eighty per cent of women in the last 2 categories were employed compared to 18.8% among those who had zero or some years of primary schooling. Living with a partner also conveyed a mean decrease in BMI of 0.3 kg/m^2^ compared to being single. Finally, mothers of babies born with a birth weight of >3,500 g had a mean BMI increase of 0.2 kg/m^2^ compared to mothers of babies with <2,500 g.

The multivariate analysis stratified by country (Table 7) shows EPBF had a significantly decreased BMI of 0.1 kg/m^2^/month for Zambian participants. South Africa had exactly opposite (of borderline significance) outcome of EPBF on mothers’ BMI. No effect from EPBF was seen in the other countries. This relationship may be partially explained by the median duration of EPBF and the proportion of women still on EPBF at week 26 at the different study sites. The median duration of EPBF was highest in Burkina Faso, Uganda and Zambia (5.8 months) and lowest in South Africa (4.9 months). At weeks 26, 27 (13.2%), 4 (0.02%), 12 (0.04%) and 33 (0.06%) were still practicing EPBF in Burkina Faso, South Africa, Uganda and Zambia, respectively. Conversely, baseline BMI was consistently and significantly associated with an increase in mothers’ subsequent BMI measures in all countries throughout the lactation period. A higher CD4 count increased BMI in Burkina Faso and Zambia, whereas an HIV stage greater than one decreased BMI in Zambia. In the 4 countries, delivering by C-section or delivering a girl was associated with mean BMI changes in different ways. In Burkina Faso, single mothers had lowered BMI, whereas in South Africa it increased, these participants being in the highest socio-economic tertile. The trial arm was not associated with any BMI change.

**Table 7:** Multivariate country-stratified analysis testing BMI change in breastfeeding mothers over the duration of extended EBF.

Adjusted Coefficient (95% CI)				
	**Burkina Faso**	**South Africa**	**Uganda**	**Zambia**
**EPBF duration**	0.0 (-0.1; 0.2)	0.1 (-0.0; 0.1)	-0.0 (-0.1; 0.1)	-0.1 (-0.1; -0.0)
**Baseline BMI**	0.9 (0.9; 1.0) §	1.0 (0.9; 1.0) §	1.0 (0.9; 1.0) §	1.0 (0.9; 1.0) §
**Mothers’ age**	-0.0 (-0.0; 0.0)	0.0 (-0.0; 0.1)	-0.00 (-0.0; 0.0)	-0.0 (-0.0; 0.0)
**CD4 count**				
<500	1	1	1	1
> = 500	0.5 (0.2; 0.8) §	0.2 (-0.1; 0.4)	0.1 (-0.1; 0.4)	0.2 (0.0; 0.4)
**Child sex**				
Male	1	1	1	1
Female	-0.4(-0.6;-0.1) §	0.5 (0.1; 0.9) §	-0.5 (-0.8; -0.2) §	-0.8 (-1.8; 0.1)
**HIV stage**				
1	1	1	1	1
>1	0.4 (-0.1; 0.8)	0.6 (-0.4; 1.6)	-0.0 (-0.1; 0.1)	-0.1 (-0.1; -0.0)
**Marital status**				
Single	1	1	1	1
Married/cohabiting	-1.0 (-1.5; -0.5) §	-	-	-
**Mode of delivery**				
Vaginal	1	1	1	1
C-section		-0.5 (-1.0; -0.1) §		0.6 (0.0; 1.2)
**Trial arm**				
lamivudine	1	1	1	1
lopinavir/ritonavir	-0.0 (-0.3; 0.3)	0.2 (-0.2; 0.6)	-0.16 (-0.3; 0.0)	-0.2 (-0.4; 0.0)
**Child birth weight**				
<2.5 kg	1	1		
> = 2.5 to 3.4 kg	0.1 (-0.4; 0.6)	-0.5 (-1.2; 0.2)	-0.1 (-0.6; 0.3)	0.3 (-0.1; 0.8)
> = 3.5 kg	0.3 (-0.3; 0.9)	0.0 (-0.7; 0.8)	0.2 (-0.7; 0.2)	0.4 (-0.2; 0.9)
**SES (tertiles)**				
Highest tertile	1			
Middle tertile	-0.3 (-0.6; 0.0)	0.0 (-0.5; 0.5)	0.2 (-0.4; 0.1)	-0.1 (-0.4; 0.1)
Lowest tertile	0.0 (-0.3; 0.4)	0.6 (0.1; 1.1) §	0.2 (-0.0; 0.5)	0.2 (-0.1; 0.6)

§ Significant results

## Discussion

Considering our results, no marked change in BMI was seen in this cohort over a 26-week period of lactation covering the EPBF period neither in the pooled analysis (Table 6) nor in the country-stratified analysis.The mean change from 26 to 50 weeks after birth was 0.7 kg/m^2^ at the paired t-test. This was also the case for changes in hemoglobin concentration. However, the thinnest mothers had the largest change, although not statistically significant. The major factor contributing to the biggest change in BMI was the mother’s own BMI after birth. The heaviest mothers gained most weight during lactation. Higher birth weights and CD4 counts correlated with some increase in BMI. Education level showed the opposite pattern, with a higher educational status being associated with a slight reduction in BMI. In the country-stratified analysis, socio-economic status was not a risk factor for BMI change, except in South Africa where high SES was associated with an increase in BMI. However, a HIV stage above 1 correlated with a small decrease in BMI in Zambia.

The magnitude of the mean BMI decrease observed in Table 2 does not seem enough important to have a clinical significance. The most important decrease (minus 1.1 kg/m^2^) was seen in Burkina Faso at week 26. The mean BMI for this country was 23.8 (95% CI: 23.2; 24.3) kg/m^2^ [data not shown]. Therefore, a decrease of 1.1 kg/m^2^ did not put a participant of this site in an underweight category. This analysis was confirmed by the findings in Table 4A where the underweight women group (<18.5 kg/m^2^) had a maximum non statistically-significant weight loss of 0.5 kg/m^2^ smaller than the overall maximum of 1.1 kg/m^2^.

To the best of our knowledge, only one study [40] has tested the dose-response relationship between breastfeeding and HIV disease progression including maternal weight loss. We also tested whether the duration of EPBF was detrimental to the BMI of HIV-infected mothers. Other studies [28,29] compared any breastfeeding group with a formula-feeding group. A Tanzanian study [40], despite its loss to follow-up, concluded that neither the modality of breastfeeding (exclusive or any), nor the duration of breastfeeding had an influence on the of mothers with HIV infection. However, this research was focused on HIV disease progression, and considered parameters that included maternal HIV viral load, CD4 count and weight loss. It recorded relative risks as association measures, making it difficult to compare with our study in terms of absolute numbers. In a randomized trial on prolonged breastfeeding and maternal mortality in Zambia [41], there seemed to be no harmful effect of breastfeeding. The study was well designed, but its main defect lies in the failure toadhere fully to random assignment by the participants, although the investigators deemed this had no significant effect on the findings. Another analysis of the same Zambian data [27] over 2 years, in which there was control for confounding, such as SES, obstetric history, season and food shortage, reported net weight gain rather than weight loss, which has also been found in undernourished lactating mothers [25,42,43]. Hartmann [43] explained this weight gain by a “homeorhetic theory of metabolic adjustment” in favor of a dominant physiological state such as lactation. This may also explain why the thinnest mothers did not lose weight during EPBF period in our cohort. However, we do not have sufficient data to address issues such as food shortages, or other practical or mental issues, that could have led to the underweight status in the first place.

Our study did not have a non-breastfeding group for comparison, and therefore cannot confirm or refute the findings from Kenya [28,29], where breastfeeding women were found that lose more weight than non-breastfeeding ones. Another study in South Africa [44] showed that breastfeeding HIV-positive mothers lost more weight than breastfeeding HIV-negative mothers. In addition to the limitations of the study (including the lack of power of the study), the design did not truly allow attribution of the weight change to the breastfeeding factor, since it did not compare breastfeeding and formula groups, nor assess a dose-response by considering the duration of breastfeeding; instead, the study compared cross-sectional data at different time points.

The different studies referred to above were not directly comparable, mainly because of differences in follow-up time. It was 24 months in the Vertical Transmission Study [14]; 20 months in the Zambian study with follow-up starting at 4 months post-partum [27]; and 6 months in the Kesho Bora trial [24]. The Kenyan studies had a number of methodological issues [45].

In an analysis of 2 sets of data from Honduras [46], EBF did not affect maternal weight loss in one study regarding 119 full-term low birth-weight infants. In the second study of 141 infants of low-income primiparous women, the EBF group lost more weight than mothers feeding their children with solid foods between 4 and 6 months of age. Being of a low income group could contribute to poor dietary intake, thereby contributing to the weight loss. The study design may not have controlled properly all potential confounders.

### Validity of our findings

This analysis was done on data collected from a large sample of HIV-positive mothers from 4 different countries in a clinical trial setting. However, the rigorous selection criteria may have resulted in a recruited a study population that was not strictly representative of the general population. Hence, the association between the HIV stage and BMI change may not reflect that in the general population of HIV-positive mothers, since only patients with CD4 counts above 350 and at stage 1 or 2 were included, and almost all of them remained at the same asymptomatic stage until the end of the study. The SES index calculation proved challenging because the study environments in the 4 countries were diverse; for this reason we built a country-specific SES since a pooled 4-country index was not meaningful. Moreover, the clinical trial context was not the routine or standard care environments for most women, which may have distorted the SES effect on BMI change. However, these issues were less likely to influence the association between EPBF duration and BMI change.

It is also possible that some reporting bias regarding our variable of interest was involved, since in some sites (e.g. Burkina Faso), the study team in charge of nutritional counseling also collected the breastfeeding data. No data was collected to validate the mothers’ reports of their own feeding practices. We dropped all subjects without breastfeeding data or with obvious inaccurate data to partially mitigate this issue.

We also failed to consider the mothers’ daily food intake and physical activities as important factors in our assessment of BMI change. Nevertheless, we believe that the nutritional, financial (transport costs) and technical support provided to all participants in the trial, combined with the relative homogeneity of our study population tended to minimize the effect of disparities in general and also those that were eventually related to food intake and physical activities.

## Conclusion

Breastfeeding did not affect the BMI in HIV-1 infected Sub-Saharan African mothers when their CD4 counts were >350 cells/μl. However, a higher baseline BMI and a CD4 count >500 cells/μl led to an increase in BMI during the EPBF period. Considering the benefits of breast milk for infants, and the recurrent results from different studies elsewhere that breastfeeding does not harm HIV-1-infected mothers, this study also supports the WHO 2016 guidelines on infant feeding, which indicates that mothers living with HIV should breastfeed for at least 12 months and up to 24 months, provided that the right treatment or prophylaxis for the infection is given where formula feeding is unsafe.
